# Composition of Microbial Oral Biofilms during Maturation in Young Healthy Adults

**DOI:** 10.1371/journal.pone.0087449

**Published:** 2014-02-04

**Authors:** Daniela Langfeldt, Sven C. Neulinger, Wieland Heuer, Ingmar Staufenbiel, Sven Künzel, John F. Baines, Jörg Eberhard, Ruth A. Schmitz

**Affiliations:** 1 Institute for General Microbiology, Christian-Albrechts-University, Kiel, Germany; 2 Department of Prosthetic Dentistry and Biomaterials Science, Hannover Medical School, Hannover, Germany; 3 Department of Conservative Dentistry, Periodontology and Preventive Dentistry, Hannover Medical School, Hannover, Germany; 4 Max-Planck Institute for Evolutionary Biology, Plön, Germany; 5 Institute for Experimental Medicine, Christian-Albrechts-University, Kiel, Germany; University Medical Center Utrecht, Netherlands

## Abstract

In the present study we aimed to analyze the bacterial community structure of oral biofilms at different maturation stages in young healthy adults. Oral biofilms established on membrane filters were collected from 32 human subjects after 5 different maturation intervals (1, 3, 5, 9 and 14 days) and the respective phylogenetic diversity was analyzed by 16S rDNA amplicon sequencing. Our analyses revealed highly diverse entire colonization profiles, spread into 8 phyla/candidate divisions and in 15 different bacterial classes. A large inter-individual difference in the subjects’ microbiota was observed, comprising 35% of the total variance, but lacking conspicuous general temporal trends in both alpha and beta diversity. We further obtained strong evidence that subjects can be categorized into three clusters based on three differently occurring and mutually exclusive species clusters.

## Introduction

High-throughput sequencing approaches have revolutionized our prospects and knowledge on microbial communities. For instance the human microbiome with its high variability between host individuals and distinct species assemblies on different parts of the human body was extensively examined [1]–[3]. Studies of these communities may elucidate interactions among microbes, as well as host-microbe interactions of ecological and clinical relevance. This may aid, e.g., the prevention of detrimental biofilms in prosthetics and dentistry. The oral microbiome is one of the most diverse of the human body. More than 700 species are reported to colonize multiple niches [4]–[6], including the soft tissue surfaces and the sub- and supragingival surfaces of the teeth, as a consequence of different environmental conditions (e.g., nutrients or pH) [7], [8]. While the majority of the oral community plays an important role in preserving the oral and systemic health [6], [9]–[12], pathogens are also included at a low percentage. As a consequence of environmental changes, host response and disturbance of microbial homeostasis, an increase of the pathogenic community might promote the development of oral diseases by leading to inflammation and infection [8], [13]. Even though periodontal diseases are initiated by polymicrobial infection, several bacterial species are commonly linked to particular oral diseases. The oral microbiome is also associated with systemic diseases [14]–[17], including for instance cardiovascular disease and atherosclerosis [14], [17]. Recently, we demonstrated that even experimentally induced gingivitis - a low-level inflammation in response to bacterial biofilm formation after a few days of suspended oral hygiene - triggers a systemic increase of surrogate markers of atherosclerotic plaque development [18]. However, the changes from health to disease as well as the mechanisms responsible for increased occurrence of pathogens are poorly understood. Likewise, studies of the human microbiome revealed that even healthy individuals could differ extremely in their bacterial community structure in same habitats, with much of this inter-individual difference remaining unexplained [2]. Thus, more insight into “healthy” consortia in oral biofilms is crucial for understanding development, prevention and treatment of oral diseases.

To evaluate potential shifts in bacterial biofilm composition during maturation and due to different bacterial colonizer pools in various hosts, we investigated oral bacterial colonization profiles close to the teeth in 32 young healthy adults (both male and female) after 5 different maturation intervals between 1 and 14 days, by 16S rDNA amplicon sequencing using a high-throughput sequencing approach.

## Materials and Methods

### Human Samples

All procedures related to subjects were approved by ethics committee of Hannover Medical School, registered at the International Clinical Trials Register Platform of the WHO (ID: DKRS00003366) and follow the guidelines of the Declaration of Helsinki. All participants gave written informed consent and were recently involved in our earlier study that investigated the systemic effects of experimental gingivitis.

### Criteria for Participation

Human subjects agreeing following criteria were selected: (a) 20–30 years of age, (b) non-smokers, (c) no clinical signs of gingival inflammation, (d) no probing pocket depth >3 mm at any site and (e) no alveolar bone loss. Criteria for exclusion were: (a) systemic diseases (e.g. diabetes mellitus), (b) pregnancy or breastfeeding, (c) history of drug abuse, (d) allergic diathesis, (e) medications (e.g. antibiotics) within 3 months before entering the study, (f) untreated carious lesions and/or insufficient restorations, implants, crowns and (g) mouth breathing.

### Biofilm Formation and Sampling

In order to obtain sufficient plaque material for sequencing maxillary and mandibulary impressions were obtained and used to fabricate individual acrylic splints. Membrane filters (MilliporeExpress® PLUS) were attached to the splints next to the teeth and gingiva by surgical sutures. In each case four membranes on the upper and four on the lower jaw on buccal and oral sides of the teeth rows. Participants were advised to carry these splints for the time intervals 1, 3, 5, 9 and 14 days and to store them in provided humidified chambers during the meals and daily oral hygiene procedures. On every sampling day all 8 filters were removed from splints, washed with sterile PBS and immediately frozen in liquid nitrogen until further processing.

### Nucleic Acid Isolation

Genomic DNA of dental plaque was isolated from a pool of 2 different membrane filters using the RTP® Bacteria DNA Mini Kit (STRATEC Molecular, Birkenfeld/Germany) following the manufacturer`s instruction (protocol 4).

### PCR Amplification and Deep Sequencing Analysis

The hypervariable regions 1 to 2 (V1–2) of the 16S rRNA gene was amplified from isolated genomic DNA using universal primer Pyro_27F (5′*CTATGCGCCTTGCCAGCCCGC*TCAGTCAGAGTTTGATCCTGGCTCAG3′) and barcoded reverse primer 338R (5′*CGTATCGCCTCCCTCGCGC*CATCAGXXXXXXXXXXCATGCTGCCTCCCGTAGGAGT3′). The primer contained the 454 Life Sciences Adaptor B (forward) and A (reverse) denoted by italics and the underlined sequences represent the broadly conserved bacterial primers 27F and 338R. A two-base linker sequence (TC/CA) and four-base key (TCAG) were added as recommended by Roche (454). A unique 10mer multiplex identifier (designated X) was added to every reverse primer to tag each PCR product. Template DNA (100 ng) was added to a 25 µL PCR reaction mix including Phusion Hot Start DNA Polymerase (Finnzymes, Vantaa/Finland). Cycling conditions started with an initial denaturation step for 30 s at 98°C, followed by 35 cycles of 9 s at 98°C, 30 s at 55°C and 30 s at 72°C and ended with a final extension for 10 min at 72°C. All reactions were performed in duplicates and combined after PCR. Amplicons were size-checked, purified with the MiniElute Gel Extraction Kit (Qiagen, Hilden/Germany) and quantified with the Quant-iT dsDNA Broad-Range Assay Kit (Invitrogen, Darmstadt/Germany) using a NanoDrop 3300 fluorometer. Equimolar amounts of purified PCR product were pooled and further purified using Agencourt AMPure XP (Beckman Coulter, Krefeld/Germany). A sample of each library was run on an Agilent Bioanalyzer prior to emulsion PCR and sequencing as recommended by Roche. Amplicon libraries were sequenced on a 454 GS-FLX using Titanium sequencing chemistry (Roche, Mannheim/Germany).

### Sequence Processing

All steps of sequence processing were conducted with the program mothur v1.27.0 [19]. Raw sequencing data in standard flowgram format were first assigned to samples according to their multiplex identifiers (MIDs) with the forward primer sequence removed. Only reads between 360 and 720 flows, with a maximum homopolymer count of 8 and no differences to MIDs/forward primer were retained (command *trim.flows*). Data were denoised (*shhh.flows*) and kept if their mean phred score [20], [21] was ≥25 after denoising (*trim.seqs*). Sequences were aligned (*align.seqs*) to the SILVA reference alignment based on the SSURef database (v102) of bacterial sequences [22] provided on http://www.mothur.org/wiki/Silva_reference_files. The alignment was optimized by deleting the shortest and longest 2.5% of the sequences, respectively (*screen.seqs*), followed by removal of gap-only columns as well as columns containing missing data at both ends of the alignment (*filter.seqs*). The resulting alignment had 696 positions with sequence lengths ranging from 239 to 315 nucleotides. In order to further reduce PCR/pyrosequencing errors, reads with one mismatch to a more abundant sequence were merged with the latter (*pre.cluster*). Chimeras were eradicated employing mothur’s implementation of Perseus [23] (*chimera.perseus*, *remove.seqs*). Initial sequence classification was conducted by a Bayesian approach [24] using a k-mer size of 8 and a bootstrap threshold of 60% (*classify.seqs*). The RDP [25] reference taxonomy (http://www.mothur.org/w/images/4/4a/Trainset7_112011.pds.zip) was used with reference sequences trimmed to the V1–V2 primer region to improve accuracy of the classification [cf. [26]. The dataset was screened for chloroplast sequences to be removed (*remove.lineage*) as these sequences would obviously have originated from vegetable food rather than from bacteria. A random subset of 1,042 sequences per sample (corresponding to the smallest number of reads above 1,000 across samples) was generated (*sub.sample*) to eliminate bias due to unequal sampling effort. A coverage of about 1,000 sequences per sample is suggested as a good balance between number of samples and depth of sampling [27]. Three samples (p35d01, p35d03, and p36d09) containing fewer sequences (855, 594, and 957, respectively) were kept in the dataset with reservation. The aligned and subsampled dataset was used to compute a distance matrix (*dist.seqs*) for binning sequences into operational taxonomic units (OTU) by average neighbor clustering (*cluster.split*). Based on a 97% similarity threshold (roughly corresponding to species level distinction) 1,779 OTUs were identified. A sample-by-OTU table was generated (*make.shared*) which for each sample states the number of sequences belonging to a certain OTU. This table was the basis for subsequent bioinformatic analyses. OTUs were classified according to the CORE reference database, a phylogenetically curated 16S rDNA database of the core oral microbiome [28], as described above for the RDP reference taxonomy (*classify.seqs*, followed by *classify.otu*).

### Sequence Abundance Plots

All downstream computations were performed in R v2.15.2 [29] with custom scripts (available from the authors on request). Initial characterization of bacterial community composition was performed by summarizing OTU abundances at genus level. Genus-level abundances were normalized by dividing their number by the total number of sequences per sample and visualized as stacked bar plots.

### Alpha Diversity Analysis

Effective OTU richness (also known as Shannon numbers equivalent, ^1^D) [30], [31] was calculated from the sample-by-OTU table using the R package vegan [32]. Initial data visualization was performed with the R package lattice [33]. The study design corresponds to a mixed-effects model in which the fixed effect “Day” (i.e., maturation time of the biofilm) is nested within the random effect “Subject”. To estimate the effect of maturation time on effective OTU richness, ^1^D was fitted to “Day” in a generalized linear mixed model using function *gamm* of R package mgcv [34] with either random intercept or random intercept and slope. Models were compared by means of the Akaike Information Criterion (AIC) with restricted maximum likelihood estimation as described by Zuur *et al.* [35].

### Beta Diversity Analysis

Redundancy Analysis (RDA) was used to explore the extent of change in OTU composition (also known as turnover) in oral microbial communities within subjects over time. OTU abundances were subjected to Hellinger transformation in order to make them compatible with RDA. This transformation downweights highly abundant OTUs, providing a good compromise between linearity and resolution [36]. RDA was thus performed on Hellinger distances between samples (i.e. Euclidean distance of Hellinger-transformed data). The model was evaluated with vegan’s function (*rda*) and consisted of the fixed effect “Day” and the “Subject” effect as conditioning term: OTU count data ∼ “Day” + Condition (“Subject”). The model was tested for significance with function *anova.cca* using 1,000 random permutations stratified within subjects. Homoscedasticity of the fixed effect was assessed using vegan’s *betadisper* function, followed by *permutest* with permutations stratified within subjects.

### Consensus Clustering of Species

OTU abundances were normalized by total counts per sample and summarized at species level (henceforward called ‘species abundances’ for simplicity). Taxa that could not be distinguished at the species level or with terms “uncultured” or “unclassified” added to their names were removed from the dataset. Species relative abundances were used for the resulting sample-by-species table which was subjected to Principal Component Analysis (PCA) after Hellinger transformation. Based on this ordination configuration, weighted-average scores of species were calculated by vegan’s (*wascores*) function for projection of species points into the PCA space of samples (scaling 1). Consistent clusters of species were determined by average-linkage clustering based on Spearman rank correlations of relative species abundances. Cluster number and robustness were assessed by consensus clustering [37] with package ConsensusClusterPlus [38] based on 1,000 random species subsamples (drawing without replacement) with a sampling proportion of 0.8. The optimal number of clusters was determined by assessing the relative change in the area under the consensus cumulative distribution function [cf. [37]. Robust cluster members were defined as species whose item consensus was ≥0.6 for the cluster they were assigned to and ≤0.4 for any other cluster. The multivariate coefficient of variation (RV) was calculated for each pair of robust species clusters based on rank-transformed species abundances. Clusters of robust species were projected as “spider” graphs into the PCA space of the samples.

### Consensus Clustering of Subjects

Partitioning of human subjects in relation to the clusters of robust species was assessed as follows. For each subject, mean relative species abundances were calculated across all time points. The resulting subject-by-species table was subjected to PCA after Hellinger transformation. The matrix of subjects’ PCA scores (scaling 1) was subjected to consensus clustering as described above with 10,000 random subject subsamples, using Partitioning Around Medoids [39] as a robust grouping algorithm. The resulting subjects’ partitioning was evaluated by Analysis of Similarities (ANOSIM) on Euclidean distances of the Hellinger-transformed subject-by-species table with 100,000 random permutations.

### 3D Visualization

A 3D plot of combined clustering and ordination data was produced in kinemage format [40] using the in-house developed R package R2Kinemage and displayed in KiNG v2.21 [41].

### Nucleotide Sequence Accession Numbers

Sequence data were submitted to the NCBI (National Center for Biotechnology Information) Sequence Read Archive under accession no. SRP027013. Table S1 of file S1 lists multiplex identifiers and corresponding information for samples in the respective sequence libraries.

## Results

### Characterization of Bacterial Biofilm Composition during Maturation

**Figure 1:**
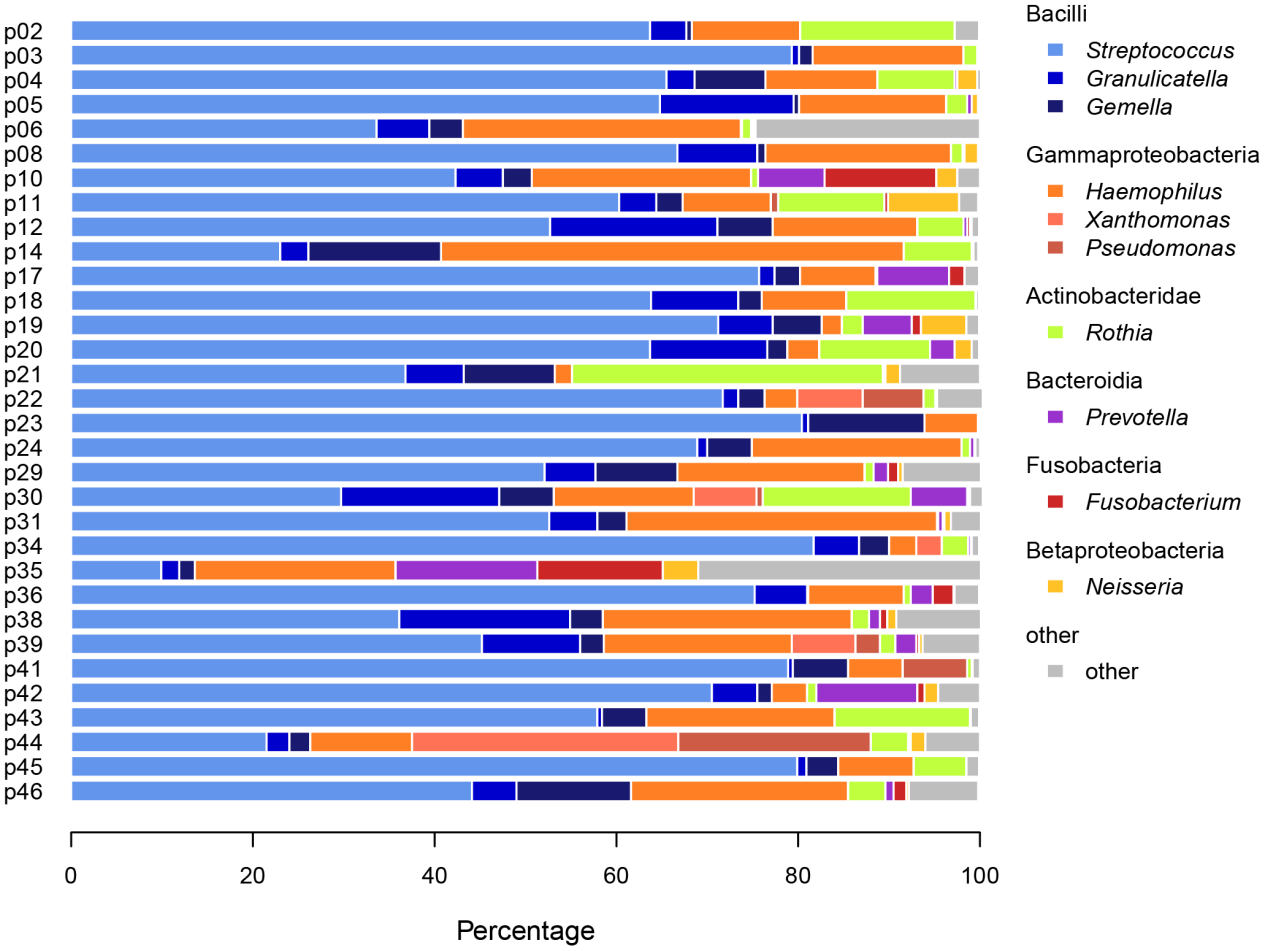
Mean bacterial composition of oral biofilms over time in individual subjects. Bacterial communities of oral biofilms in 32 different human subjects were analyzed by 16S rDNA amplicon deep sequencing (V1–V2 region). Taxa (mean relative abundance across subjects ≥1%) are shown at class and genus levels.

Oral biofilm formation on membranes close to the gingiva was monitored in 32 healthy adults over 14 days. Aiming to examine the bacterial biofilm composition during the establishment, five different time points in biofilm formation were collected (day 1, 3, 5, 9 and 14). A total of 160 biofilm samples were analyzed after DNA extraction and amplifying the hypervariable region V1–V2 of the 16S rDNA by 454 pyrosequencing. 1,042 sequences per sample were retained after random subsampling, binned into a total of 1,779 operational taxonomic units (OTUs) of which about only 15 were highly abundant across all samples (see Tables S2 and S3 for a list of raw OTU counts and corresponding taxonomy in file S1, respectively). Variability of sample diversity was high, ranging from 7 to 130 OTUs, distributed over 8 major phyla/candidate divisions (Firmicutes, Proteobacteria, Actinobacteria, Bacteroidetes, Fusobacteria, Spirochaetae, SR1 and TM7). The phyla could further be subdivided into 15 identified classes (Bacilli, Clostridia, Erysipelotrichi, Negativicutes, α-, β-, γ-, ε-Proteobacteria, Actinobacteridae, Coriobacteridae, Bacteroidia, Flavobacteria, Sphingobacteria, Fusobacteria, Spirochaetes) (see Table S4 in file S1). Comparison of the bacterial composition of all individuals over all time points by relative abundance plots revealed highly variable colonization profiles (Fig. 1).

One subject (p35) exhibited a conspicuously low level of *Streptococcus* in combination with elevated levels of *Prevotella*, *Fusobacterium* and other minor taxa over time in comparison with the other subjects (Fig. 1). Since measures strongly deviating from the mean can have adverse effects on regression-type analyses as performed here, we chose to exclude subject p35 from subsequent analyses.

As a measure of alpha diversity, we employed effective OTU richness ^1^D. Initial data visualization did not reveal conspicuous general trends in ^1^D with time (Fig. S1 in file S1). To check for a possible less obvious temporal pattern, we fitted a generalized linear mixed model with “Day” as fixed effect. Because residuals showed clear deviation from normality (validated by visual inspection of a quantile-quantile plot), a gamma distribution with log link was used to model ^1^D. Comparison by AIC suggested the random intercept model (the intercept of the regression line may vary between subjects) as the best choice. While the model was valid (no conspicuous residual patterns were observed), the effect of variable “Day” was not significant (p = 0.13), thus no time-related change in alpha diversity of oral microbiota was observed.

In beta diversity analysis, Hellinger-transformed OTU counts were subject to RDA with “subjects” as conditioning term to account for inter-individual variation. The effect of “Day” on the change of individual OTU abundances between samples of the same subject was not significant (p≈0.1). Accordingly, adjusted R^2^ (a measure for the variance explained by the RDA model) was only 0.5%. Hence there was no obvious and consistent time-related change in relative OTU abundances within subjects. In contrast, a comparatively large portion of variance (35%) was attributed to the conditioning term “Subject”, arguing for a large inter-individual difference in microbial composition.

### Evidence of Mutual Exclusions between Different Bacterial Complexes

**Figure 2:**
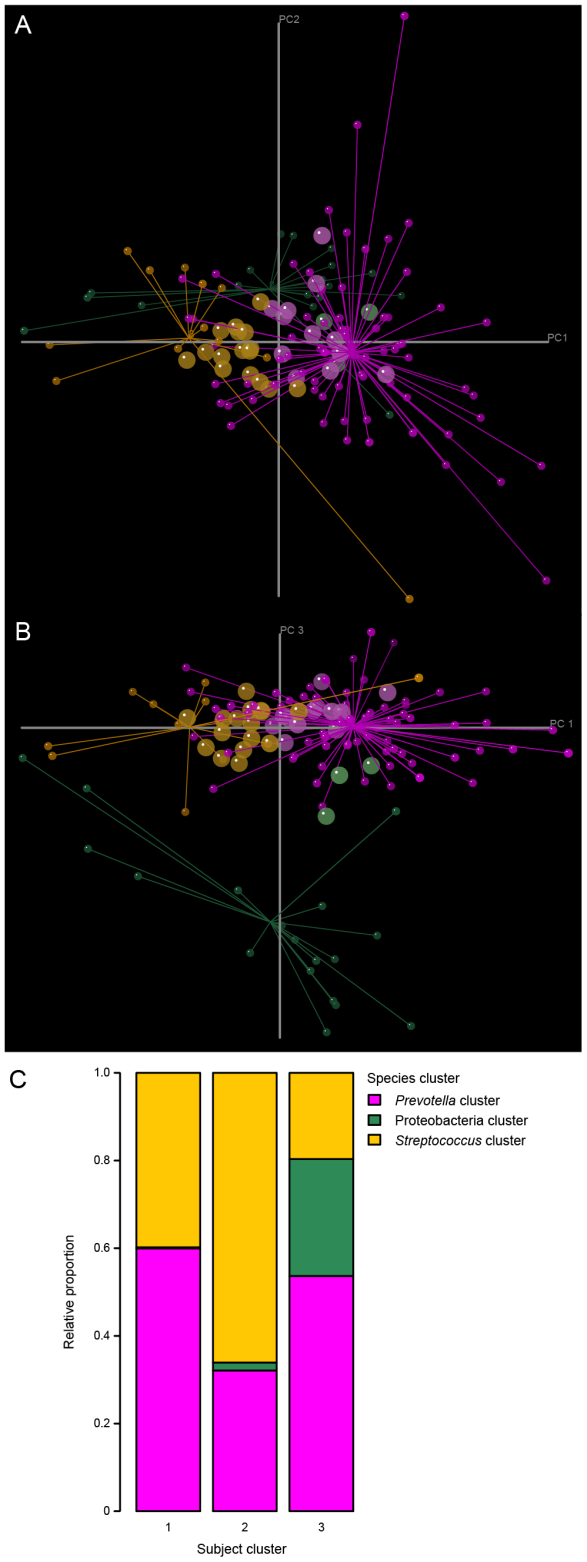
Species and subject clusters. Combined consensus clustering and ordination (PCA) of robust species and human subjects. (A) First (PC1) and second (PC2) axis and (B) first (PC1) and third axis (PC3) of the ordination space of individual subject samples are shown. Species data points (small spheres) are projected into the ordination space as weighted averages and grouped into three clusters according to species consensus clustering: “*Prevotella* cluster” (magenta), “*Streptococcus* cluster” (orange), “Proteobacteria cluster” (green). Large spheres represent the centroids of individual sample points for each human subject, color-coded according to the result of subject consensus clustering. (C) Relative species abundances in subject clusters. Color coding of species clusters is analogous to (A) and (B).

Since alpha and beta diversity analysis did not show any significant time-related response in our data, we concentrated on evaluating correlations between individual species across all data points. For this purpose, we employed Spearman rank correlation for species abundances combined with consensus clustering - a simple and straightforward method of inferring robust clusters by resampling - to obtain robust results. A combined consensus clustering and unconstrained ordination analysis (Principal Component Analysis) revealed three clusters of species mutually excluding each other across subjects and time points (Fig. 2A, B; see also Fig. S2 in file S1 for an interactive version of this image with additional display options such as taxon names, subject codes and individual subject time points; see Table S5 for a list representation of species clusters in file S1). These three clusters of species differed in their relative abundances (averaged over time) in three corresponding subject clusters (Fig. 2C). Separation of the latter was corroborated by a fairly large ANOSIM statistic (R = 0.54) with high statistical significance (p<10^−5^). The magenta-colored species cluster was dominated by different *Prevotella* species (“*Prevotella* cluster”), the green one contained β- and γ-Proteobacteria (“Proteobacteria cluster”) and the orange-colored cluster mainly contained *Streptococcus* species (“*Streptococcus* cluster”). The “*Prevotella* cluster” and the “*Streptococcus* cluster” showed a strong multivariate correlation (RV = 0.3; p<0.01) and mutually exclude each other. A weak but still significant multivariate correlation (RV = 0.07; p<0.01) was observed between the “Proteobacteria cluster” and the contrasting “*Prevotella* cluster”. No significant correlation could be observed between the “Proteobacteria cluster” and the “*Streptococcus* cluster”. Fig. 2C shows the relative species abundances (averaged over time and subjects) in the 3 detected subject clusters. Subject cluster 1 was dominated by the “*Prevotella* cluster” (60%) and “*Streptococcus* cluster” (40%). In the second subject cluster the “*Streptococcus* cluster” dominated (66%), followed by members of the “*Prevotella* cluster” (32%) and a minority of the “Proteobacteria cluster” (2%). The third subject cluster exhibited the largest occurrence of the “Proteobacteria cluster” (27%), while the “*Prevotella* cluster” represented about half of the counts (54%) and the “*Streptococcus* cluster” was least abundant (20%).

## Discussion

Oral microbial profiles in health and disease have been investigated in numerous *in vivo* studies [11], [12], [42]. In this respect, several bacterial species have been shown to be directly linked to specific diseases [12], [43], [44]. However, the transitional stages from health to disease as well as the mechanisms responsible for appearance of those pathogens are poorly understood. Thus, elucidating “healthy” complex consortia of oral biofilms in the course of time is crucial. In the present investigation we addressed this essential aspect by analyzing microbial composition of oral biofilms of 32 healthy human subjects during maturation over 14 days at 5 different time points.

### Analysis of Oral Biofilms Over Time Revealed High Inter-individual Variation

Within the 16S rDNA amplicon sequencing approach of oral biofilms we analyzed a highly diverse dataset with more than 1,700 different OTUs at the 97% similarity level, although only 15 of them were highly abundant across all samples. The number of OTUs within each sample ranged from only 7 up to 130 consequently already suggesting a large variation between individuals. Further evidence for large inter-individual differences in microbial composition was obtained by RDA. This is in accordance with previous findings that the oral community is especially diverse in contrast to other body habitats, e.g. the vagina [2], [45]. The consistency of our 16S rDNA profiles of oral biofilms is comparable to the keratinized gingiva baseline at the phylum level in healthy subjects as reported by the Human Microbiome Project [46]. Comparison to a lower level is impaired by differences due to methods and approaches used, e.g. primer set, database, sequences per sample and number of subjects.

We did not detect any systematic time-related patterns, neither in alpha nor in beta diversity. This suggests that both, the cumulative number of OTUs as well as their individual turnover in a subject, is influenced more by random (or unaccounted/unknown) effects than by time in the course of biofilm formation between 24 h and 14 days. These findings are in agreement with previous reports that propose a high variability in the oral microbiome between individuals [11], [47] as well as a minimal temporal variability in individuals [45]. The Human Microbiome Project Consortium further reported on within-subject stability of the human microbiome by sampling an additional time point after approximately 220 days. They hypothesized that the stable individual microbial community may be another feature of the human microbiome specifically associated with health [2]. This assumption is supported by previous studies of microbial diversities of different body habitats, which has been linked to different diseases [48]–[50]. For instance the complex stool communities were markedly reduced in specific gut diseases and bacterial vaginosis is associated with a high microbial diversity, whereas healthy vaginal sides harbored simpler communities [2]. Our study of oral biofilms in healthy subjects over time correlates favorably with these findings and further supports the idea of stable complex oral microbiota in health that obviously differ among subjects.

### Mutual Exclusion of Bacterial Consortia and their Assignment to Corresponding Subject Clusters

The most remarkable result obtained from our data set is the clustering of species into three groups that mutually exclude each other and differ in their relative abundances in three corresponding subject clusters (Fig. 2, Fig. S2 in file S1). To our best knowledge, this is the first study providing culture-independent evidence for species clusters in the human oral microbiome.

In particular, the strongest exclusionary relationship was observed between the “*Streptococcus* cluster” and “*Prevotella* cluster”. Mutualistic interactions as well as interspecies competition among microbial partnerships are often triggered simultaneously by several members of oral biofilms and are in the focus of current research [51]–[56]. A possible mechanism for such an “interspecies defense” might be the release of bacteriocins produced by several streptococci as well as by numerous Gram-negative bacteria of the oral cavity. These effectively act against a variety of further bacterial members within this habitat [57]–[60]. In addition, hydrogen peroxide produced by oral streptococci has been shown to inhibit the growth of other members of the oral cavity [54], [61]. Certain oral streptococci have been shown to negatively impact biofilm formation of *Porphyromonas gingivalis* [62], [63]. While *P. gingivalis* has not been detected in our dataset, this species is known to coaggregate with *Prevotella intermedia* [64], which is indeed a member of the “*Prevotella* cluster”. Moreover, the “*Prevotella* cluster” included other *Porphyromonas* spp. Hence, our study provides additional evidence for the reciprocal exclusion of streptococci and *Porphyromonas* spp.

Our results of cluster analyses are further in agreement with previous findings obtained by a co-culture experiment with cluster-representative species. Herein, Stingu *et al*. [65] observed significant bactericidal effects of oral streptococci, in particular *S. sanguinis* and *S. anginosus*, on *P. intermedia*. Further studies of Stingu and collaborators showed that the development of periodontitis is associated with an increased colonization of two *Prevotella* species (*P. intermedia* and *P. nigrescens)* as well as a reduced establishment of *S. sanguinis* [66], [67].

Despite of the high inter-individual microbial variation in oral biofilms, our results revealed the existence of various colonizer pools in different hosts. We assume that even in health, the oral bacterial community features a broad potential compositional spectrum. The obtained composition of the oral biofilm of a certain individual may be indicative for this person’s ability to resist pathogens or disease susceptibility. Although all species in the identified clusters are members of the oral commensally community, there is evidence that some of these microorganisms are associated with gingivitis [68]–[70]. These include Gram-positive species (e.g., *Streptococcus* ssp. and *Parvimonas micra*), as well as numerous Gram-negative species (e.g., *Campylobacter gracilis, Fusobacterium nucleatum, Prevotella intermedia* and *Veillonella*). Remarkably, all those bacteria are present in the identified “*Prevotella* cluster”. Moreover, another study [71] detected *Capnocytophaga* ssp., which is also a member of the “*Prevotella* cluster”, on the onset of gingivitis and identified *Prevotella* spp. in areas with established gingivitis. On the other hand, species that are associated with periodontal health - such as primary or early colonizers (e.g., *Streptococcus* and *Gemella*) [9], [72] - have been predominantly found in the identified “*Streptococcus* cluster”. Since we know from our earlier study with the same subject cohort [18] that the human subjects responded differently to an experimentally induced gingivitis in terms of inflammation severity (Eberhard, unpublished data), we hypothesize a potential association of species cluster prevalence with disease susceptibility and inflammatory condition of human subjects. However, future investigations are mandatory to address this aspect.

Another conspicuity is the third subject cluster that consists of only three test persons. This cluster was shown to host a substantial proportion of the “Proteobacteria cluster”, which exhibits another - albeit weaker - contrasting relationship with the “*Prevotella* cluster”. The role of this cluster consisting primarily of aerobic β*-* and γ-Proteobacteria remains to be elucidated. However, in sufficient quantity it appears to replace the “*Streptococcus* cluster”.

### Optimized Study Design and Application of Meta’omics Approaches may Reveal Trends Related to Time and Susceptibility to Inflammation

Due to the high inter-individual variability in microbial composition, an optimized study design is crucial to observe a potential time effect in oral biofilm formation. Even though our subject group was quite homogenous in terms of age and health status, several systemic and external factors such as diet, gender, hormone status and stress known to have an impact on the oral microbiome were not controlled for in the present study. This contributed to the observed highly diverse colonization profiles. A subsequent investigation with a stricter control of the subject set-up, optimally over periods longer than 14 days, may reveal clearer temporal trends in alpha diversity. Since a potential correlation between the oral microbiome and inflammatory diseases such as gingivitis might exist at the strain or even at the genomic/metabolic level, this correlation may not be reflected by 16S rDNA based phylogenetic analysis even after optimization of the experimental set up. Consequently, more advanced tools such as shotgun metagenomics or metatranscriptomics [73] should be preferentially employed to uncover such relationships.

In summary, our results provide evidence that, despite high microbial diversity in individuals, different consortia composition of oral microbiota exist in healthy hosts. External factors as well as physical predisposition of the host might be responsible and determine the structure of these consortia. Moreover first indications were obtained that community structures might be indicative for host’s disease susceptibility.

## Supporting Information

File S1 Table S1: Multiplex identifiers and corresponding information on samples in the respective sequence libraries. Table S2: OTU counts in subjects over time. Table S3: OTU taxonomy. Table S4: Bacterial abundances at class-level in subjects over time. Table S5: List of species names in species clusters. Fig. S1: Alpha diversity analysis. Effective OTU richness ^1^D is shown within individual subjects over time. Fig. S2: Kinematic image representation of the combined consensus clustering and ordination (PCA) of robust species and human subjects shown in Fig. 2. Additional display options comprise species names, subject codes, individual subject time points, and means of time points over all subjects. Note: The free KiNG display software (Kinemage, Next Generation; http://kinemage.biochem.duke.edu/software/king.php) is needed to view the image.
(ZIP)

## References

[1] Gevers D, Knight R, Petrosino JF, Huang K, McGuire AL, et al.. (2012) The Human Microbiome Project: A Community Resource for the Healthy Human Microbiome. Plos Biology 10. 10.1371/journal.pbio.1001377 PMC3419203 22904687

[2] Huttenhower C, Gevers D, Knight R, Abubucker S, Badger JH, et al (2012) Structure, function and diversity of the healthy human microbiome. Nature 486: 207–214. 22699609 10.1038/nature11234 PMC3564958

[3] Methe BA, Nelson KE, Pop M, Creasy HH, Giglio MG, et al (2012) A framework for human microbiome research. Nature 486: 215–221. 22699610 10.1038/nature11209 PMC3377744

[4] Paster BJ, Boches SK, Galvin JL, Ericson RE, Lau CN, et al (2001) Bacterial diversity in human subgingival plaque. Journal of Bacteriology 183: 3770–3783. 11371542 10.1128/JB.183.12.3770-3783.2001 PMC95255

[5] Parahitiyawa NB, Scully C, Leung WK, Yam WC, Jin LJ, et al (2010) Exploring the oral bacterial flora: current status and future directions. Oral Diseases 16: 136–145. 19627515 10.1111/j.1601-0825.2009.01607.x

[6] Aas JA, Paster BJ, Stokes LN, Olsen I, Dewhirst FE (2005) Defining the normal bacterial flora of the oral cavity. Journal of Clinical Microbiology 43: 5721–5732. 16272510 10.1128/JCM.43.11.5721-5732.2005 PMC1287824

[7] Rasiah IA, Wong L, Anderson SA, Sissons CH (2005) Variation in bacterial DGGE patterns from human saliva: over time, between individuals and in corresponding dental plaque microcosms. Archives of Oral Biology 50: 779–787. 15970209 10.1016/j.archoralbio.2005.02.001

[8] Avila M, Ojcius DM, Yilmaz O (2009) The Oral Microbiota: Living with a Permanent Guest. DNA and Cell Biology 28: 405–411. 19485767 10.1089/dna.2009.0874 PMC2768665

[9] Socransky SS, Haffajee AD (1992) The bacterial etiology of destructive periodontal disease: current concepts. Journal of Periodontology 63: 322–331. 10.1902/jop.1992.63.4s.322 1573546

[10] Zaura E, Keijser BJF, Huse SM, Crielaard W (2009) Defining the healthy “core microbiome” of oral microbial communities. Bmc Microbiology 9. 10.1186/1471-2180-9-259 PMC2805672 20003481

[11] Bik EM, Long CD, Armitage GC, Loomer P, Emerson J, et al (2010) Bacterial diversity in the oral cavity of 10 healthy individuals. Isme Journal 4: 962–974. 20336157 10.1038/ismej.2010.30 PMC2941673

[12] Kumar PS, Griffen AL, Moeschberger ML, Leys EJ (2005) Identification of candidate periodontal pathogens and beneficial species by quantitative 16S clonal analysis. Journal of Clinical Microbiology 43: 3944–3955. 16081935 10.1128/JCM.43.8.3944-3955.2005 PMC1233920

[13] Pennisi E (2005) A mouthful of microbes. Science 307: 1899–1901. 15790840 10.1126/science.307.5717.1899

[14] Beck JD, Offenbacher S (2005) Systemic effects of periodontitis: Epidemiology of periodontal disease and cardiovascular disease. Journal of Periodontology 76: 2089–2100. 10.1902/jop.2005.76.11-S.2089 16277581

[15] Paju S, Scannapieco FA (2007) Oral biofilms, periodontitis, and pulmonary infections. Oral Diseases 13: 508–512. 17944664 10.1111/j.1601-0825.2007.1410a.x PMC2258093

[16] Scannapleco FA, Genco RJ (1999) Association of periodontal infections with atherosclerotic and pulmonary diseases. Journal of Periodontal Research 34: 340–345. 10685358 10.1111/j.1600-0765.1999.tb02263.x

[17] Seymour GJ, Ford PJ, Cullinan MP, Leishman S, Yamazaki K (2007) Relationship between periodontal infections and systemic disease. Clinical Microbiology and Infection 13: 3–10. 17716290 10.1111/j.1469-0691.2007.01798.x

[18] Eberhard J, Grote K, Luchtefeld M, Heuer W, Schuett H, et al.. (2013) Experimental Gingivitis Induces Systemic Inflammatory Markers in Young Healthy Individuals: A Single-Subject Interventional Study. Plos One 8. 10.1371/journal.pone.0055265 PMC3567060 23408963

[19] Schloss PD, Westcott SL, Ryabin T, Hall JR, Hartmann M, et al (2009) Introducing mothur: Open-source, platform-independent, community-supported software for describing and comparing microbial communities. Applied and Environmental Microbiology 75: 7537–7541. 19801464 10.1128/AEM.01541-09 PMC2786419

[20] Ewing B, Hillier L, Wendl MC, Green P (1998) Base-calling of automated sequencer traces using phred. I. Accuracy assessment. Genome Research 8: 175–185. 9521921 10.1101/gr.8.3.175

[21] Ewing B, Green P (1998) Base-calling of automated sequencer traces using phred. II. Error probabilities. Genome Research 8: 186–194. 9521922

[22] Pruesse E, Quast C, Knittel K, Fuchs BM, Ludwig W, et al (2007) SILVA: a comprehensive online resource for quality checked and aligned ribosomal RNA sequence data compatible with ARB. Nucleic Acids Research 35: 7188–7196. 17947321 10.1093/nar/gkm864 PMC2175337

[23] Quince C, Lanzen A, Davenport RJ, Turnbaugh PJ (2011) Removing noise from pyrosequenced amplicons. BMC Bioinformatics 12: 38. 21276213 10.1186/1471-2105-12-38 PMC3045300

[24] Wang Q, Garrity GM, Tiedje JM, Cole JR (2007) Naïve Bayesian classifier for rapid assignment of rRNA sequences into the new bacterial taxonomy. Applied and Environmental Microbiology 73: 5261–5267. 17586664 10.1128/AEM.00062-07 PMC1950982

[25] Cole JR, Wang Q, Cardenas E, Fish JA, Chai B, et al (2009) The Ribosomal Database Project: improved alignments and new tools for rRNA analysis. Nucleic Acids Research 37: D141–145. 19004872 10.1093/nar/gkn879 PMC2686447

[26] Werner JJ, Koren O, Hugenholtz P, DeSantis TZ, Walters WA, et al (2012) Impact of training sets on classification of high-throughput bacterial 16S rRNA gene surveys. ISME Journal 6: 94–103. 21716311 10.1038/ismej.2011.82 PMC3217155

[27] Hamady M, Knight R (2009) Microbial community profiling for human microbiome projects: Tools, techniques, and challenges. Genome Research 19: 1141–1152. 19383763 10.1101/gr.085464.108 PMC3776646

[28] Griffen AL, Beall CJ, Firestone ND, Gross EL, Difranco JM, et al (2011) CORE: a phylogenetically-curated 16S rDNA database of the core oral microbiome. PLoS One 6: e19051. 21544197 10.1371/journal.pone.0019051 PMC3081323

[29] R Core Team (2012) R: A language and environment for statistical computing. Vienna, Austria: R Foundation for Statistical Computing.

[30] Jost L (2006) Entropy and diversity. Oikos 113: 363–375.

[31] Jost L (2007) Partitioning diversity into independent alpha and beta components. Ecology 88: 2427–2439. 18027744 10.1890/06-1736.1

[32] Oksanen J, Blanchet FG, Kindt R, Legendre P, Minchin PR, et al.. (2012) vegan: Community ecology package. R package version 2.1–20/r2309 ed.

[33] Sarkar D (2008) Lattice: Multivariate data visualization with R. New York: Springer.

[34] Wood SN (2011) Fast stable restricted maximum likelihood and marginal likelihood estimation of semiparametric generalized linear models. Journal of the Royal Statistical Society: Series B (Statistical Methodology) 73: 3–36.

[35] Zuur AF, Ieno EN, Walker NJ, Saveliev AA, Smith GM (2009) Mixed effects models and extensions in ecology with R; Gail M, Krickeberg K, Samet JM, Tsiatis A, Wong W, editors: Springer.

[36] Legendre P, Gallagher ED (2001) Ecologically meaningful transformations for ordination of species data. Oecologia 129: 271–280. 28547606 10.1007/s004420100716

[37] Monti S, Tamayo P, Mesirov J, Golub T (2003) Consensus clustering: A resampling-based method for class discovery and visualization of gene expression microarray data. Machine Learning 52: 91–118.

[38] Wilkerson M (2011) ConsensusClusterPlus. R package version 1.10.0 ed.

[39] Kaufman L, Rousseeuw PJ (1990) Finding groups in data: An introduction to cluster analysis: Wiley-Interscience.

[40] Richardson DC, Richardson JS (1992) The kinemage: A tool for scientific communication. Protein Science 1: 3–9. 1304880 10.1002/pro.5560010102 PMC2142077

[41] Chen VB, Davis IW, Richardson DC (2009) KING (Kinemage, Next Generation): A versatile interactive molecular and scientific visualization program. Protein Science 18: 2403–2409. 19768809 10.1002/pro.250 PMC2788294

[42] Tanner A, Maiden MFJ, Macuch PJ, Murray LL, Kent RL (1998) Microbiota of health, gingivitis, and initial periodontitis. Journal of Clinical Periodontology 25: 85–98. 9495607 10.1111/j.1600-051x.1998.tb02414.x

[43] Ximenez-Fyvie LA, Haffajee AD, Socransky SS (2000) Comparison of the microbiota of supra- and subgingival plaque in health and periodontitis. Journal of Clinical Periodontology 27: 648–657. 10983598 10.1034/j.1600-051x.2000.027009648.x

[44] Socransky SS, Smith C, Haffajee AD (2002) Subgingival microbial profiles in refractory periodontal disease. Journal of Clinical Periodontology 29: 260–268. 11940147 10.1034/j.1600-051x.2002.290313.x

[45] Costello EK, Lauber CL, Hamady M, Fierer N, Gordon JI, et al (2009) Bacterial Community Variation in Human Body Habitats Across Space and Time. Science 326: 1694–1697. 19892944 10.1126/science.1177486 PMC3602444

[46] Segata N, Haake SK, Mannon P, Lemon KP, Waldron L, et al.. (2012) Composition of the adult digestive tract bacterial microbiome based on seven mouth surfaces, tonsils, throat and stool samples. Genome Biology 13. 10.1186/gb-2012-13-6-r42 PMC3446314 22698087

[47] Nasidze I, Li J, Quinque D, Tang K, Stoneking M (2009) Global diversity in the human salivary microbiome. Genome Research 19: 636–643. 19251737 10.1101/gr.084616.108 PMC2665782

[48] Turnbaugh PJ, Hamady M, Yatsunenko T, Cantarel BL, Duncan A, et al (2009) A core gut microbiome in obese and lean twins. Nature 457: 480–U487. 19043404 10.1038/nature07540 PMC2677729

[49] Qin JJ, Li RQ, Raes J, Arumugam M, Burgdorf KS, et al (2010) A human gut microbial gene catalogue established by metagenomic sequencing. Nature 464: 59–U70. 20203603 10.1038/nature08821 PMC3779803

[50] Fredricks DN, Fiedler TL, Marrazzo JM (2005) Molecular identification of bacteria associated with bacterial vaginosis. New England Journal of Medicine 353: 1899–1911. 16267321 10.1056/NEJMoa043802

[51] Periasamy S, Kolenbrander PE (2009) *Aggregatibacter actinomycetemcomitans* Builds Mutualistic Biofilm Communities with *Fusobacterium nucleatum* and *Veillonella* Species in Saliva. Infection and Immunity 77: 3542–3551. 19564387 10.1128/IAI.00345-09 PMC2738031

[52] Periasamy S, Kolenbrander PE (2009) Mutualistic Biofilm Communities Develop with Porphyromonas gingivalis and Initial, Early, and Late Colonizers of Enamel. Journal of Bacteriology 191: 6804–6811. 19749049 10.1128/JB.01006-09 PMC2772475

[53] Jakubovics NS, Kolenbrander PE (2010) The road to ruin: the formation of disease-associated oral biofilms. Oral Diseases 16: 729–739. 20646235 10.1111/j.1601-0825.2010.01701.x

[54] Jakubovics NS, Gill SR, Vickerman MM, Kolenbrander PE (2008) Role of hydrogen peroxide in competition and cooperation between Streptococcus gordonii and Actinomyces naeslundii. Fems Microbiology Ecology 66: 637–644. 18785881 10.1111/j.1574-6941.2008.00585.x PMC2820160

[55] Kuramitsu HK, He XS, Lux R, Anderson MH, Shi WY (2007) Interspecies interactions within oral microbial communities. Microbiology and Molecular Biology Reviews 71: 653-+. 10.1128/MMBR.00024-07 PMC2168648 18063722

[56] Kreth J, Merritt J, Shi WY, Qi FX (2005) Competition and coexistence between *Streptococcus mutans* and *Streptococcus sanguinis* in the dental biofilm. Journal of Bacteriology 187: 7193–7203. 16237003 10.1128/JB.187.21.7193-7203.2005 PMC1272965

[57] van der PloegJR (2005) Regulation of bacteriocin production in Streptococcus muttans by the quorum-sensing system required for development of genetic competence. Journal of Bacteriology 187: 3980–3989. 15937160 10.1128/JB.187.12.3980-3989.2005 PMC1151730

[58] Hyink O, Wescombe PA, Upton M, Ragland N, Burton JP, et al (2007) Salivaricin A2 and the novel lantibiotic salivaricin B are encoded at adjacent loci on a 190-kilobase transmissible megaplasmid in the oral probiotic strain Streptococcus salivarius K12. Applied and Environmental Microbiology 73: 1107–1113. 17194838 10.1128/AEM.02265-06 PMC1828679

[59] Wescombe PA, Heng NCK, Burton JP, Chilcott CN, Tagg JR (2009) Streptococcal bacteriocins and the case for *Streptococcus salivarius* as model oral probiotics. Future Microbiology 4: 819–835. 19722837 10.2217/fmb.09.61

[60] Kaewsrichan J, Douglas CWI, Nissen-Meyer J, Fimland G, Teanpaisan R (2004) Characterization of a bacteriocin produced by *Prevotella nigrescens* ATCC 25261. Letters in Applied Microbiology 39: 451–458. 15482437 10.1111/j.1472-765X.2004.01608.x

[61] Holmberg K, Hallande.Ho (1973) Production of bactericidal concentrations of hydrogen peroxide by *Streptococcus sanguis*. Archives of Oral Biology 18: 423–&. 10.1016/0003-9969(73)90167-2 4515970

[62] Wang BY, Wu J, Lamont RJ, Lin XH, Xie H (2009) Negative Correlation of Distributions of *Streptococcus cristatus* and *Porphyromonas gingivalis* in Subgingival Plaque. Journal of Clinical Microbiology 47: 3902–3906. 19846640 10.1128/JCM.00072-09 PMC2786655

[63] Christopher AB, Arndt A, Cugini C, Dayey ME (2010) A streptococcal effector protein that inhibits *Porphyromonas gingivalis* biofilm development. Microbiology-Sgm 156: 3469–3477. 10.1099/mic.0.042671-0 PMC7336479 20705665

[64] Kamaguchi A, Nakayama K, Ohyama T, Watanabe T, Okamoto M, et al (2001) Coaggregation of *Porphyromonas gingivalis* and *Prevotella intermedia* . Microbiology and Immunology 45: 649–656. 11694077 10.1111/j.1348-0421.2001.tb01298.x

[65] Stingu CS, Rodloff AC (2011) Interaction between oral streptococci and two *Prevotella* species: an in vitro study. Clinical Microbiology and Infection 17: S508.

[66] Stingu C-S, Schaumann R, Jentsch H, Eschrich K, Brosteanu O, et al.. (2013) Association of periodontitis with increased colonization by Prevotella nigrescens. Journal of investigative and clinical dentistry 4. 10.1111/j.2041-1626.2012.00129.x 22767485

[67] Stingu CS, Eschrich K, Rodloff AC, Schaumann R, Jentsch H (2008) Periodontitis is associated with a loss of colonization by Streptococcus sanguinis. Journal of Medical Microbiology 57: 495–499. 18349371 10.1099/jmm.0.47649-0

[68] Theilade E, Wright WH, Jensen SB, Loe H (1966) Experimental gingivitis in man. II. A longitudinal clinical and bacteriological investigation. Journal of periodontal research 1. 10.1111/j.1600-0765.1966.tb01842.x 4224181

[69] Kremer BHA, Loos BG, van der Velden U, van Winkelhoff AJ, Craandijk J, et al (2000) *Peptostreptococcus micros* smooth and rough genotypes in periodontitis and gingivitis. Journal of Periodontology 71: 209–218. 10711611 10.1902/jop.2000.71.2.209

[70] Macuch PJ, Tanner ACR (2000) Campylobacter species in health, gingivitis, and periodontitis. Journal of Dental Research 79: 785–792. 10728981 10.1177/00220345000790021301

[71] Mombelli A, Lang NP, Burgin WB, Gusberti FA (1990) Microbial changes associated with the development of puberty gingivitis. Journal of Periodontal Research 25: 331–338. 2148945 10.1111/j.1600-0765.1990.tb00924.x

[72] Paster BJ, Falkler WA, Enwonwu CO, Idigbe EO, Savage KO, et al (2002) Prevalent bacterial species and novel phylotypes in advanced noma lesions. Journal of Clinical Microbiology 40: 2187–2191. 12037085 10.1128/JCM.40.6.2187-2191.2002 PMC130824

[73] Segata N, Boernigen D, Tickle TL, Morgan XC, Garrett WS, et al.. (2013) Computational meta/’omics for microbial community studies. Molecular Systems Biology 9. 10.1038/msb.2013.22 PMC4039370 23670539

