# Supplementary figures and images for: Composition of Microbial Oral Biofilms during Maturation in Young Healthy Adults
Source: PLoS One. 2014 Feb 4;9(2):e87449. doi: 10.1371/journal.pone.0087449 (PMC3913613; doi:10.1371/journal.pone.0087449)

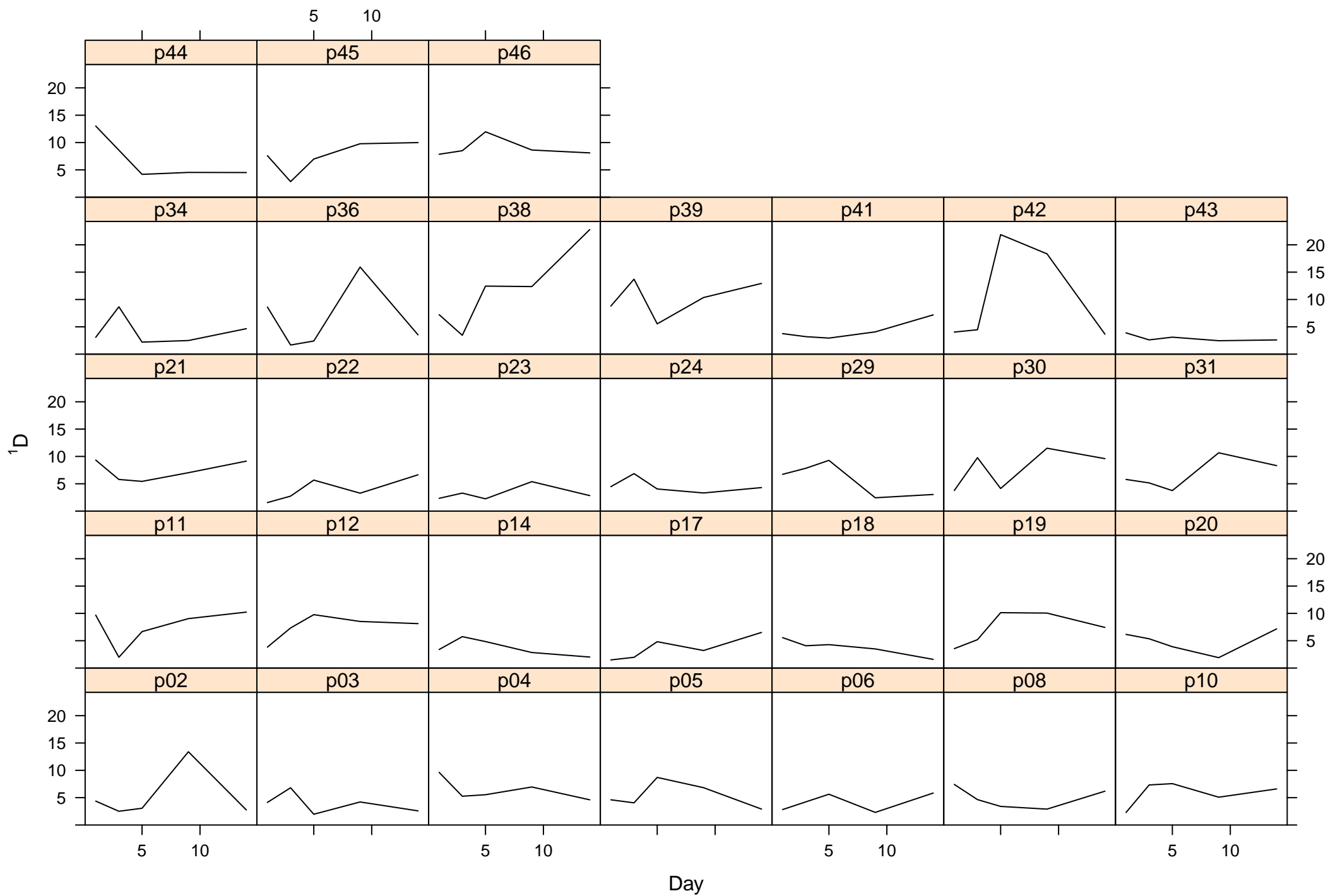

Supplement: File S1 — Table S1: Multiplex identifiers and corresponding information on samples in the respective sequence libraries. Table S2: OTU counts in subjects over time. Table S3: OTU taxonomy. Table S4: Bacterial abundances at class-level in subjects over time. Table S5: List of species names in species clusters. Fig. S1: Alpha diversity analysis. Effective OTU richness 1D is shown within individual subjects over time. Fig. S2: Kinematic image representation of the combined consensus clustering and ordination (PCA) of robust species and human subjects shown in Fig. 2. Additional display options comprise species names, subject codes, individual subject time points, and means of time points over all subjects. Note: The free KiNG display software (Kinemage, Next Generation; http://kinemage.biochem.duke.edu/software/king.php) is needed to view the image. (ZIP) [file pone.0087449.s001.zip › Figure_S1.pdf]
